# Towards Consensus on Good Practices for the Use of New Technologies for Intervention and Support in Developmental Dyslexia: A Delphi Study Conducted among Italian Specialized Professionals

**DOI:** 10.3390/children8121126

**Published:** 2021-12-03

**Authors:** Maria Luisa Lorusso, Francesca Borasio, Martina Da Rold, Andrea Martinuzzi

**Affiliations:** 1 Unit of Child Psychopathology, Scientific Institute IRCCS E. Medea, 23842 Bosisio Parini, Italy; francesca.borasio@lanostrafamiglia.it; 2 Department of Psychology, Catholic University of the Sacred Heart, 20123 Milano, Italy; 3 Epilepsy and Neurophysiology Unit, Scientific Institute IRCCS E. Medea, 31015 Conegliano, Italy; martina.darold@lanostrafamiglia.it (M.D.R.); andrea.martinuzzi@lanostrafamiglia.it (A.M.)

**Keywords:** developmental dyslexia, new technologies, intervention, augmented reality, virtual reality, good practices, Delphi method, rehabilitation

## Abstract

The use of new technologies for intervention in developmental dyslexia is steadily growing. In order to better understand the needs, the expectations, and the attitudes of Italian expert health professionals concerning such technologies, a national survey was conducted applying the Delphi methodology. Ad-hoc questionnaires were sent out to a group of eighteen experts over three successive rounds, and anonymously collected responses were aggregated and shared with the group after each round, aiming to reach a consensus on the proposed response. The goal was to define a series of statements that could form the basis for international “good practices” in the use of technologies for intervention to support dyslexia in children and adolescents. In the first round, the experts’ general opinions were collected with both multiple choice and open questions, and in the second round consensus was assessed on a series of statements based on the first replies. The cut-off of 75% consensus on each statement was reached after three rounds. Fifteen experts completed all the rounds of the process, and a final version of the statements regarding good practice in the use of technologies for dyslexia could be defined.

## 1. Introduction

Recent decades have seen a change in educational and clinical settings under the influence of information and communications technology (ICT). ICTs are technological systems (e.g., hardware devices and software applications) that allow the production, storage, communication, and sharing of information [1,2]. The various types of technology currently used in education and clinical activity meet the diverse needs of the users, from teachers and clinicians or parents, to students and patients. Categorization into low-tech, mid-tech, and high-tech is based on the usability, development, and practicality of the technologies; it ranges from highlighters or adapted pencils, to computers, mobile devices, and artificial intelligence applications [3,4].

In recent years, high-tech technologies were adopted in studies regarding learning disorders, for instance text-to-speech and speech-to-text applications for the empowerment of reading and writing skills [5]. Technologies may support the learning process, giving more opportunity to practice, providing immediate feedback and an individualized and flexible learning environment [4,6].

Augmented reality and virtual reality are two types of technology used in education and intervention projects.

Augmented reality (AR) provides an interactive experience with the real world, where objects in the world are enhanced by perceptual information generated by the computer [7]. The first applications of AR in the education setting, designed twenty-five years ago, were mainly based on head-mounted displays and were too expensive and complex to be used in extensive field experiments. The second generation of AR, from 2010 to 2019, was mainly integrated in mobile devices, with the start of a rather controversial research line focused on the use of smart glasses, attempting to empower people’s lives by placing information just before their eyes. These changes resulted in reduced costs and increased usability. The third generation of the present day is further enriched by artificial intelligence (AI) and AR-based web, providing various types of users with more autonomy, social integration, motivation, and enjoyment [4]. Studies have shown that AR may offer several advantages when used in educational settings [7,8]. For instance, AR helps university students build laboratory skills and positive attitudes relating to physics laboratories [9], in addition to aiding reading comprehension tasks through motivating and interesting games that promote problem solving, exploration, and socialization [10].

Virtual reality (VR) is an interactive simulation created with computer hardware and software to generate a fully immersive experience, an environment that appears similar to the real world [11,12]. There are different types of VR that range from fully immersive to non-immersive according to the degree of isolation from the physical surroundings while the user interacts with the virtual environment [13]. In recent years, VR has been used in the context of health care and has been proven to represent an alternative possibility for neurorehabilitation [14,15,16]. Similarly to AR, VR also allows the creation of tailor-made training programs and the adaptation of the rehabilitation process to each patient’s specific needs [14].

Several studies underlined the effects of technology on students with and without special needs, such as students with learning disorders [5,6,17].

Dyslexia is a neurodevelopmental disorder characterized by difficulties in accurate or fluent word recognition and spelling, which occurs despite typical intelligence, adequate educational resources, motivation, and an absence of neurological or psychiatric problems [18,19].

Developmental dyslexia, according to national clinical guidelines [20,21], is diagnosed from the end of the second year of schooling (when children are between seven and eight years old) and appropriate treatment can be provided at either public or private centers. Nonetheless, intervention may be provided even before that age in the case of known risk factors (e.g., family history or concomitant language disorders with phonological impairment) or in the prospect of prevention. The guidelines also specify that treatment can be intensive and should last between a few weeks to three/four months, possibly provided in subsequent cycles [20,21].

Some studies have pointed out that the treatment of dyslexia may require intensive training, explicit instructions for the exercises, and single person or small group implementation [22,23]. Standard treatment methods use a classic paper and pencil format, but children may find such exercises boring or too repetitive. Technology represents great support for dyslexic children in order to achieve their educational goals [6,16,17,24]. A recent review has reported improvements in reading, writing, and calculation skills following the use of technological tools in children with learning disorders [5].

Various studies using Wii-based, or computer, games have shown that a short and intensive treatment with an action-based video game, rather than the training of phonological or orthographic skills, may improve reading abilities in dyslexic children, specifically visual-attention abilities, spatial cognition, auditory spatial attention, and response time speed [25,26,27,28,29]. A recent study using VR technology for the rehabilitation of reading deficits in dyslexia found that a virtual environment may represent a valid approach to improve attentional skills [16].

The applicability, usability, and practicality of these technological tools are important elements to check when deciding to implement a technological treatment program. Moreover, in order to produce positive effects in reading and writing skills in children with dyslexia, it is crucial to tailor technological tools to the specific characteristics of children. When designing ICT tools, one should take into consideration, for example, font types and sizes or screen colors that facilitate reading [5,30]. Even though all of the ICT tools are considered to be useful, there are still few studies on the use and effects of artificial intelligence, augmented reality, and virtual reality [6].

The main goal of the present study is to formulate a series of statements that could form the basis for international “good practices” on the use of technologies in the treatment of dyslexia or specific reading disorders in children and adolescents. The study is situated in the context of the European ForDys-Var Erasmus+ project (https://fordysvar.eu/ (accessed on 20 November 2021)), whose objective is to improve learning in people with dyslexia through technology, specifically virtual reality and augmented reality. The project is being conducted in collaboration with three different European countries, namely Italy, Romania, and Spain.

During the present study, an online survey was conducted in order to reach a consensus on recommendations using the Delphi method. The Delphi method, originally created in the 1950s by the Rand corporation [31], is a group facilitation process aiming to obtain a consensus regarding the opinions of experts through multiple rounds of questionnaires. After each round, anonymous responses are aggregated and shared with the group. Before starting such a multistage process in order to combine opinion into group consensus, a panel of carefully selected participants must be identified. This group of experts should demonstrate involvement and expertise in the field related to the research topic [31,32]. Although it is crucial to have adequate panel members to form a heterogeneous group, there is no agreement regarding the optimal size of a Delphi panel, with several studies including fewer than 20 participants (e.g., [33,34]). The panel of experts receives an initial Delphi questionnaire that may include open-ended questions, qualitative comments are encouraged. Then, comments from the whole group are sent to the participants through a second questionnaire, and feedback is requested to show the comparison between the individual’s ratings and the whole distribution. Statements can be modified considering the feedback and a third questionnaire is thus formulated. This process is repeated until an adequate degree of consensus is reached among the group.

## 2. Materials and Methods

### 2.1. Questionnaire Design

Following Murphy and colleagues [35], a three-round Delphi survey was conducted for this study. In particular, we used the digital method, called the e-Delphi method, consisting of an online survey platform to collect data [36]. An agreement of at least 75% on each question was proposed to define a consensus. The questionnaire included both statements on the use of ICT in general, and more specific questions on AR and VR, as the study aimed to collect opinion and consensus on the general use of technology for intervention addressing reading disorders. Specifically, VR and AR were chosen to represent some of the most cutting-edge technologies, which were also developed and employed in the ForDysVar project.

### 2.2. Participants

The online questionnaire was sent to a group of 18 professionals, including 13 psychologists, 3 child neuropsychiatrists, and two speech-and-language pathologists who are among the most recognized experts in Italy in the field of intervention for dyslexia and who were known by the authors to have at least some experience with intervention tools based on new technologies. All of the selected experts are part of at least one of the main Italian scientific associations involved in the study and clinical practice of reading disorders: AIRIPA (Associazione Italiana per la Ricerca e l’Intervento in Psicopatologia dell’Apprendimento—Italian Society for Research and Intervention in the Psychopathology of Learning processes) and AID (Associazione Italiana Dislessia—Italian Dyslexia Association). Some of the experts had personally contributed as scientific consultants to the development and/or validation (not-for-profit) of technological tools for intervention in neurodevelopmental disorders (mainly computerized games aimed to improve phonological skills, memory, or attention). None of them had any conflict of interest. The experts were invited by email. In the email text, the title, and the instructions of the online questionnaire, it was clearly stated that the goal of the survey was to define a set of international “good practices” for the use of technologies for the treatment and support of developmental dyslexia.

### 2.3. Procedure

#### 2.3.1. Data Collection

Data from the three rounds of the e-Delphi survey were collected between September 2020 and February 2021. Before starting the online survey, participants were informed (both in an email and in the online questionnaire) that their responses would be recorded in a completely anonymous form with no possibility to retrieve the respondents’ identities. They were further informed that the completion of the questionnaire implied that they agreed to the collection and the processing of their responses in this anonymous form, as well as to their use for scientific purposes and future publications. All the questionnaires were implemented using the Google Form application, with obligatory responses to each of the closed questions. For each round, three emails were sent to the whole panel over a period of 2–3 weeks, and the collection of the responses was closed 3 weeks after the last email. During each round, the experts received the link to access the results from the previous round as reported on the Google Form page (after response collection had been closed).

#### 2.3.2. Round 1

The questionnaire for Round 1 consisted of 21 questions about technology applied to dyslexia (12 multiple choice questions and nine open-ended questions, see Table 1). The questions and the response options were formulated based on previous literature as to represent the most controversial issues for clinical use. Since the literature did not always provide specific information, some of the questions were based on the authors’ direct clinical experience with technology for the rehabilitation of reading disorders, or on their own opinions, always providing answers that could either confirm or deny their hypotheses. The panel could provide comments and suggestions for each of the questions (an open-ended question for comments was provided after each of the question in the online survey form). At the end of this stage, the replies were analyzed in order to formulate the statements that were to be rated by the same group of experts in the second step of the Delphi procedure.

#### 2.3.3. Round 2

After the completion of Round 1, 39 statements based on the previous survey were sent to the same group of experts. Open space was added after each statement to suggest possible improvements. The experts were asked to express their degree of agreement with each statement on a 5-level, Likert-like scale from 1 (strongly disagree) to 5 (strongly agree). The intermediate level 3 (labelled as “I do not know”) was used to express a lack of knowledge or expertise. An overall 75% group consensus was the target required to determine a positive outcome and stop the process.

#### 2.3.4. Round 3

**Figure 1:**
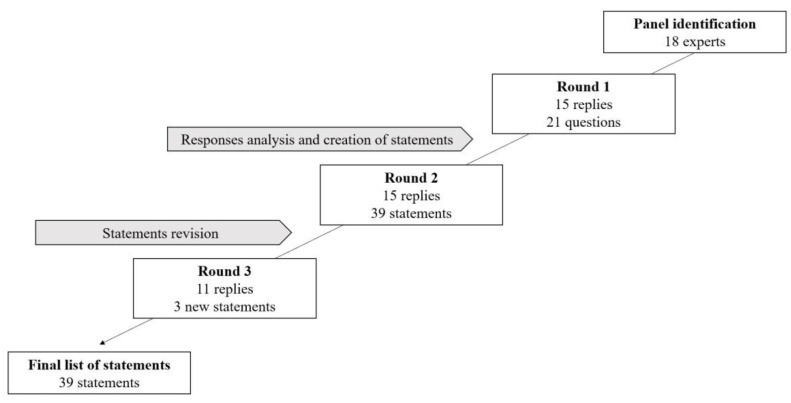
Flowchart of the Delphi process.

The questionnaire was further revised following Round 2, providing alternative wordings for the statements that had not reached the consensus cut-off of 75% in Round 2. Participants were requested to express their degree of agreement on the same scale used in Round 1, with the new statements only. This was sent to all panel members and their replies were collected. Figure 1 shows the flowchart of the Delphi process.

### 2.4. Data Analysis

Data collected in the three rounds of the Delphi survey were analyzed qualitatively. Questions of Round 2 and Round 3 provided answers that could be led back to an ordinal scale with five levels, possible replies were “strongly disagree, disagree, do not know, agree, and strongly agree”. The answer “I do not know” was initially assigned an intermediate value of 3 in equivalent scores, but when it was observed (from the open-ended responses) that its use was limited to experts who declared to have no or little knowledge of the device being judged, it was decided to consider it as a null reply and not to include it in the calculation of the degree of agreement. Thus, the scale was treated as a four-level scale, with the possible answers “strongly disagree, disagree, agree, and strongly agree”. Agreement was thus calculated as the percentage of the ratings above 3 (4 = agree, 5 = strongly agree) on the total number of responses, excluding 3 (= I do not know).

## 3. Results

### 3.1. Round 1

**Figure 2:**
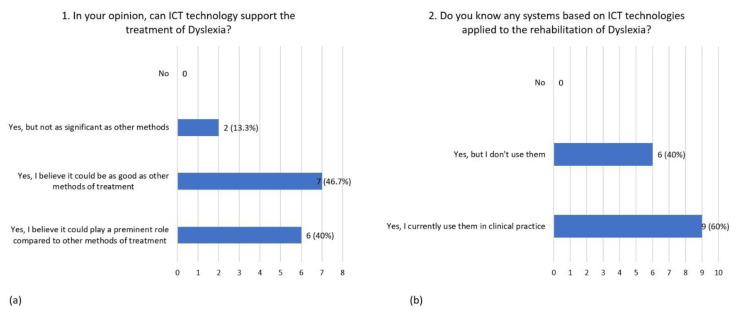
Distribution of responses to question 1 (**a**) and question 2 (**b**) on information and communications technology (ICT).

The responses collected in the first survey are presented below. Fifteen experts out of 18 completed the survey. All the respondents declared that information and communications technology (ICT) can support the treatment of dyslexia. In particular, 46.7% indicated that it could be as good as other methods, 40% indicated that it could play a preeminent role compared to other intervention methods, and 13% declared that its contribution could not be as significant as other methods (Figure 2a).

The experts declared to know some systems based on ICT technologies applied on the rehabilitation of dyslexia A total of 60% would use them in clinical practice, and 40% would not use them (Figure 2b). Specifically, experts using ICT are familiar with different kinds of software and systems widely used in Italy, and other software involving the stimulation of phonological, lexical, sublexical reading-related processes, visual recognition of graphemes, spelling, and memory.

**Figure 3:**
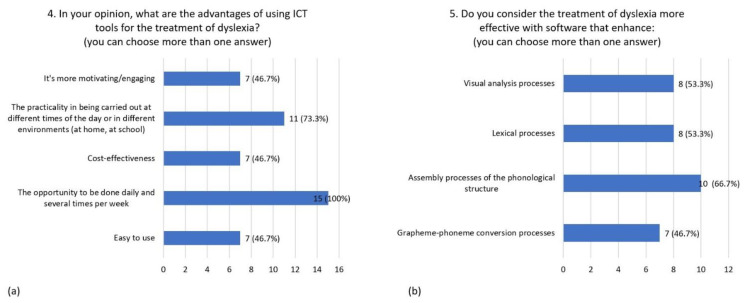
Distribution of responses to question 4 (**a**) and question 5 (**b**).

The advantage of using ICT tools for intervention in dyslexia is considered to lie in the ease of use (46.7%), the possibility of an intensive use (100%), cost-effectiveness (46.7%), the possibility to use them in different environments and at different times of the day (73.3%), and their motivating and engaging characteristics (46.7%) (Figure 3a). The treatment of dyslexia is considered to be more effective if based on software improving phonological assembly processes (66.7%), lexical processes (53.3%), visual analysis processes (53.3%), and grapheme–phoneme conversion processes (46.7%) (Figure 3b).

**Figure 4:**
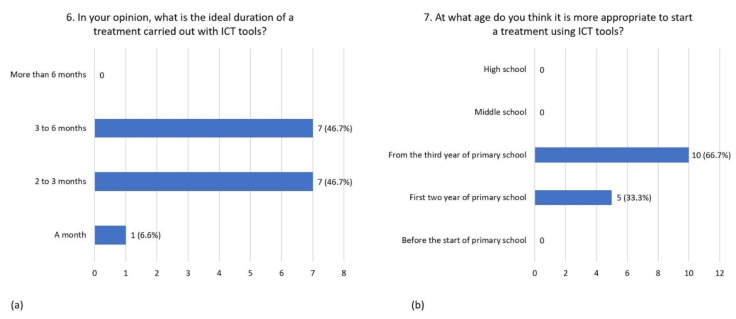
Distribution of responses to question 6 (**a**) and question 7 (**b**).

As to the question concerning optimal treatment duration, 46.7 % believe that the ideal treatment duration is from 2 to 3 months, 46.7% from 3 to 6 months, and only 6.6% indicated a one-month-duration (Figure 4a). The most appropriate age to start a treatment using ICT tools was considered to be during the first two years of primary school (66.7%) or from the third year of primary school on (33.3%) (Figure 4b).

**Figure 5:**
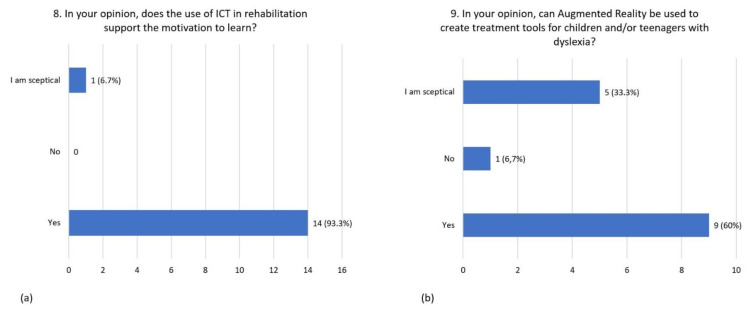
Distribution of responses to question 8 (**a**) and question 9 (**b**).

Almost all respondents declared that the use of ICT tools in treatment supports the child’s motivation to learn (93.3%), while the remaining participants declared to be skeptical (6.7%) (Figure 5a). Augmented Reality can be appropriately used to design treatment tools for children with dyslexia for 60% of the respondents, 33.3 % of them were skeptical while 6.7 % did not agree (Figure 5b).

As to the open question concerning how AR could be used in the design of treatment tools, two experts declared that AR could be used to create a more appealing interface, for example by using voices in rehabilitation tasks that are often boring and tiring for dyslexics, within an enriched context. Other respondents suggested that AR could provide reinforcements through multimodal channels and facilitate learning through more dynamic images (for instance, AR could support mathematical learning by directly providing the formulas to be applied or facilitating the visual representation of the problem), expanding the range of learning experience proposals or amplifying the stimuli to improve deficient functions and providing prompts for the identification of difficulties or errors.

When asked to indicate from what age use of AR should be recommended, three experts replied that the ideal age is from 8 years on, three indicated the period of primary school (at the beginning or from the third grade), one respondent suggested use from 4 years on, one stated that AR could be used from the moment of diagnosis, and another suggested that the type of task should be taken into consideration. Other experts stated that among the aims of AR-based treatments could be the automation of metaphonological processes and global reading skills, the improvement of critical areas, the facilitation of the use of compensatory tools, the treatment of focused attention, and the shifting of attention, or more generally, to support learning and motivation (*n* = 2).

**Figure 6:**
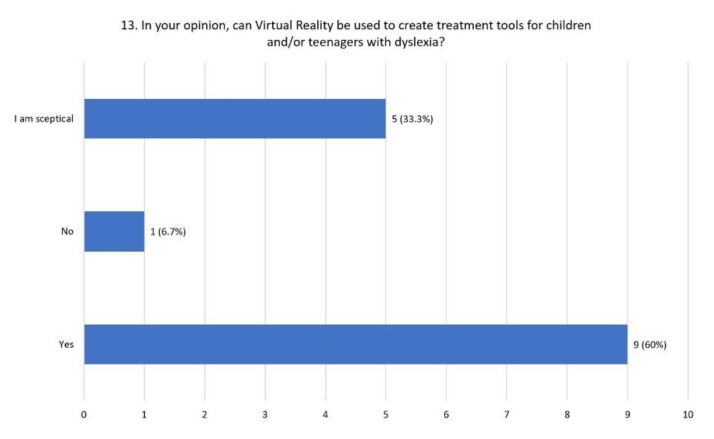
Distribution of responses to question 13.

Virtual reality can be used to create intervention tools for children with dyslexia according to 60% of the respondents, 33.3 % of them were skeptical, while 6.7 % did not agree (Figure 6).

Respondents stated that VR could be used to create learning environments to generalize acquired skills (*n* = 1) and to activate deficient skills through structured tasks in a playing situation (*n* = 1). Some experts suggested that VR could be used to enhance motor and spatial visual functions (*n* = 1); that it could be included in an integrated intervention program (*n* = 1), or in ecological contexts to facilitate learning through role playing activities (*n* = 1).

When asked from what age the use of VR could be recommended, the experts replied that the ideal age is from 8 years old or even before the age of 8 in subclinical or at-risk situations (*n* = 3). Other experts said that it could be used from primary school on (*n* = 2), from the moment of diagnosis, or depending on the type of task (*n* = 2).

Among the aims of the use of VR, the respondents listed increasing active participation and involvement (*n* = 1), activating deficient skills through exercises in the form of a game, enhancing learning, motivation and concentration, facilitating lexical access, attentional control, and perceptual discrimination.

As to the open question concerning the limitations in the use of ICT tools for the treatment of dyslexia, respondents said that is the tools are difficult to integrate into a global rehabilitation plan, may not be available at home, and require the involvement of the family if remotely operated. The level of satisfaction of the child, the risk of underutilizing the potential of digital technologies by merely proposing repetitive activities, the use of programs that engage the child through visual activities but do not really stimulate the decoding process, the possibility of feeding a dependency in subjects at risk, the reduction of social interactions and content sharing, economic issues, and the absence of mediation by the human expert (rehabilitator), were other reasons described by the experts.

**Figure 7:**
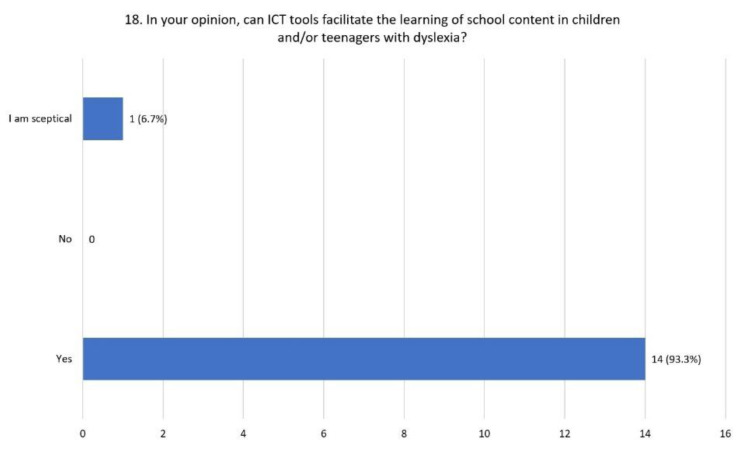
Distribution of responses to question 18.

ICT tools may facilitate the learning of school content in children with dyslexia according to 93.3% of respondents, while the remaining declared themselves to be skeptical (Figure 7).

When asked to imagine possible examples of ICT-based learning activities, experts offered different proposals, such as classes 3.0, promoting an online understanding of content research, the creation of study materials, the possibility of proposing the same multimedia content in different forms and with different degrees of complexity, encouraging creative and non-mnemonic learning, a different type of organization of activities, setting a time for a certain task, personal searches, and internet searches of study topics.

**Figure 8:**
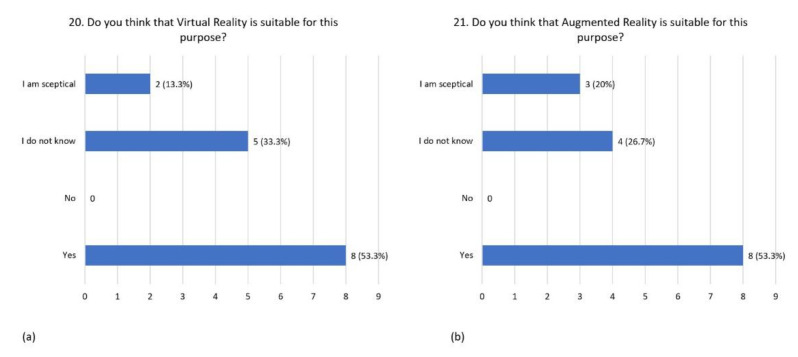
Distribution of responses to question 20 (**a**) and question 21 (**b**).

According to 53.3% of respondents, VR may be suitable for this purpose; 33.3% of them were skeptical, and 33.3% did not know (Figure 8a). Regarding AR, it may be suitable for this purpose for 53.3% of respondents, 20% of them were skeptical, and the remaining 26.7% did not know (Figure 8b).

Based on the responses collected in Round 1, presented above, a list of statements was created and included in the questionnaire used in Round 2.

### 3.2. Round 2

During Round 2 we received responses from 15 of the 18 panel members (83.33%). Two of them sent the responses at the end of Round 2, when statement 6 and statement 8 of the second survey had already been modified for Round 3. For that reason, statements 6 and 8 have 13 answers while all the remaining have 15 answers.

The statements and the degree of agreement with the different questions are presented in Table 2.

Experts provided ratings for each statement and qualitative data in the form of comments. There was a high level of agreement for most statements (mean 84.67%). Taking into account the requirement of 75% group consensus, all items achieved at least 76% agreement except for statement 6 (69.09%), statement 8 (50%), and statement 12 (70%).

Qualitative data on previous statements made it possible to understand the reasons for the low degree of agreement. Regarding statement 6 “ICT trainings should address primarily the processes involved in assembling the phonological structure of the words”, experts who expressed a low level of agreement or gave a “I do not know” reply suggested that ICT trainings may address various processes involved in reading, not only the process involved in the phonological structure of the words.

Regarding statement 8 “Grapheme-to-phoneme (and vice-versa) conversion processes may be addressed in the ICT training, but they should not be considered as prominent goals of the intervention”, three experts who expressed a low level of agreement argued that the grapheme-to-phoneme conversion process should be considered an important goal of the intervention, and another member of the panel specified that this goal depends on the child’s age.

Lastly, statement 12 “Augmented reality can be employed in the design of ICT trainings for dyslexia, but it should not play a prominent role” received an uncertain reply from five panel members (33.33%) who declared “not to know” about the topic but did not add any comment or suggestion. For that reason, the statement was not modified for Round 3 and was finally deleted.

Statement 3 “The main advantage of ICT approaches for the treatment of dyslexia is its flexibility, entailing the possibility to repeatedly propose the treatment several times per week, at the times that are more suitable for the children and their families” received a high level of agreement (87.69%) and a comment regarding the importance of the quality of the intervention, so it was decided to add a new statement in Round 3 survey to understand more about the quality and adaptation of the intervention on the level of performance.

### 3.3. Round 3

On the basis of the comments provided by the panel to the statements of Round 2, we made some further modification to the survey. The revised set of modified statements was sent to the panel for further comment, and 11 experts gave their degree of agreement to the three new statements (statement 3b added on the basis of statement 3 comments, statement 6 and 8 to replace the previous ones). Table 3 presents the degree of agreement obtained regarding the three new statements (Table 3).

All items achieved at least 82.5% agreement. According to the results of Round 3, the final agreed version of the survey was made of 39 statements, 36 belonging to the survey of Round 2 and the three new statements of Round 3.

## 4. Discussion

The aim of the present study was to define a set of statements in order to form the basis for international “good practices” in the use of technologies for intervention and support for developmental dyslexia, in the context of the European “ForDys-Var” Erasmus+ project. The study was conducted using the Delphi method [31] reaching 75% minimum agreement on all of the statements after three rounds. Eighteen psychologists, child neuropsychiatrists and speech-and-language pathologists, among the Italian most recognized experts in developmental dyslexia, took part in the study, expressing their opinions on the topic through online questionnaires.

The first round of the survey consisted of 21 questions about technology applied to dyslexia. The second survey was realized starting from the quantitative and qualitative data obtained in Round 1 and consisted of 39 statements. The panel of experts expressed their degree of agreement with each statement providing answers on a Likert-type scale with four levels, from “strongly disagree” to “strongly agree”. Two statements that did not reach 75% consensus were modified for the last survey based on the comments provided by the experts. Round 3 reached a 75% of consensus for all the statements, that were thus accepted as final recommendations.

Altogether, the recommendations emerging from the study indicate a favorable attitude by the panel members towards ICT-based intervention approaches for neurodevelopmental disorders in children, particularly dyslexia. Such practices are considered as generally effective and motivating. The experts, moreover, extended general clinical guidelines for the treatment of dyslexia to ICT treatment, recommending that it should be started before grade three and should last up to six months. Among the advantages of ICT-based approaches, the experts indicated flexibility, adaptivity (also in terms of self-adjusting algorithms), engagement, and cost-effectiveness. Among the specific advantages of AR, the experts underscored its capability to enhance specific stimulus characteristics as desired for the therapy, and its multi-sensory nature. Turning to VR, dyslexia experts appreciated its capability to create links between educational topics and real life, to sustain generalization processes, and to provide multi-sensory stimulation.

The functions listed as main targets for intervention include phonological awareness, visual abilities, lexical skills, and grapheme-to-phoneme (and vice versa) conversion.

In round 2, almost all the statements received a high level of agreement. Experts especially valued the effectiveness of ICT approaches in their integration to more traditional methods of treatment of dyslexia, increasing children’s motivation and involvement. Moreover, great importance was given to the meaningfulness of the activities to be proposed (and their ecological validity) also through a multi-sensory, multi-modal environment that could enrich the quality and quantity of stimulus-related information. Great attention is also to be paid, according to the experts, to the families’ compliance, to their possibility and capability to support the children during treatment, and to the fundamental role of constant human mediation and supervision during ICT-based activities.

Indeed, regular screen use is a fact of modern life: on average, children aged 8–12 in the United States spend 4–6 h a day using screens, while teens spend up to 9 h (data from American Academy of Child and Adolescent Psychiatry, February 2020) [37]. Media use guidelines around the world [38,39,40] encourage a correct use of screen and multimedia. For example, for preschool children, it is recommended to limit non-educational screen time to about 1 h per weekday and 3 h on weekend days; for school-age children, instead, no exact screen time limits are suggested, but guidelines highlight the importance to encourage healthy habits and limit activities that include screens. The absence of a precise cut-off for children’s screen time depends on the lack of evidence on the differential effects of different forms of screen time [40]. However, it is considered appropriate to manage screen use, discouraging media use especially during homework or meals, while encouraging meaningful screen use. As for the present study and the applications of technology addressed in it, we believe that the use of ICT for intervention and support in developmental dyslexia, and its impact in educational settings, could be considered as a healthy (and meaningful) use of screen time, but close supervision should ensure that the use of such applications is not taking the form of addiction.

Among the limitations of the present study is the low level of expertise declared by many of the panel members with regard to clinical applications of virtual reality and, particularly, of augmented reality. Indeed, for some of the statements, the experts failed to provide an agreement response, with high percentages of “I do not know” replies. In fact, the experts had been identified as prominent scholars in their discipline and experts in the use of technology for the rehabilitation of developmental dyslexia, but they were not necessarily experts in the use of advanced technologies such as virtual reality and augmented reality. This confirmed that the use of ICT for the rehabilitation of reading disorders is, at least in Italy, almost exclusively limited to more traditional forms of technology such as computerized games and exercises, and possibly text-to-speech or speech-to-text applications to support school activities, whereas newer and more advanced technologies are still rarely known and used. Despite this, we believe that the panel was representative of the state of knowledge and expertise at a national level, and that similar (and possibly less informative) results would have been obtained by contacting a different group of professionals. While it would have been possible to include a panel of ICT experts with a more technical background, this would have implied lowering the required level of knowledge on the specific characteristics of learning disorders in children, which we believed to be a necessary requirement to be able to judge the impact and the effects of technology on the children’s cognitive, psychological, and neuropsychological development.

In the context of the European “ForDys-Var” Erasmus+ project, the final set of statements will be sent to a more extended group of psychologists, child neuropsychiatrists, and speech-and-language pathologists, including teachers and school professionals of different European countries, in order to collect a consensus from a broader range of professionals and to define a set of recommendations and best practices to be shared at European level.

## Figures and Tables

**Table 1:** Round 1 questions and response options.

Questions	Answers
(1) In your opinion, can ICT technology support the treatment of Dyslexia?	-Yes, I believe it could play a preeminent role compared to other methods of treatment -Yes, I believe it could be as good as other methods of treatment -Yes, but not as significant as other methods -No
(2) Do you know any systems based on ICT technologies applied to the rehabilitation of SLDs, specific learning disorders, in particular, dyslexia?	-Yes, I currently use them in clinical practice -Yes, but I do not use them -No
(3) What kind of software/systems did you use?	Open-ended question
(4) In your opinion, what are the advantages of using ICT tools for the treatment of dyslexia? (You can choose more than one answer)	-Easy to use -The opportunity to be completed daily and several times per week -Cost-effectiveness -The practicality of being carried out at different times of the day or in different environments (at home, at school etc.) -It is more motivating/engaging
(5) Do you consider the treatment of dyslexia more effective with software that enhances? (you can choose more than one answer)	-Grapheme–phoneme conversion processes -Assembly processes of the phonological structure -Lexical processes -Visual analysis processes
(6) In your opinion, what is the ideal duration of treatment carried out with ICT tools?	-A month -2 to 3 months -3 to 6 months -More than 6 months
(7) At what age do you think it is more appropriate to start treatment using ICT tools?	-Before the start of primary school -First two years of primary school -From the third year of primary school -Middle school -High school
(8) In your opinion, does the use of ICT in rehabilitation support the motivation to learn?	-Yes -No -I am skeptical
(9) In your opinion, can augmented reality be used to create treatment tools for children and/or teenagers with dyslexia?	-Yes -No -I am skeptical
(10) If yes, how?	Open-ended question
(11) If yes, from what age?	Open-ended question
(12) If yes, with what aim?	Open-ended question
(13) In your opinion, can virtual reality be used to create treatment tools for children and/or teenagers with dyslexia?	-Yes -No -I am skeptical
(14) If yes, how?	Open-ended question
(15) If yes, from what age?	Open-ended question
(16) If yes, with what aim?	Open-ended question
(17) What limits do you see in the use of ICT tools for the treatment of dyslexia?	Open-ended question
(18) In your opinion, can ICT tools facilitate the learning of school content in children and/or teenagers with dyslexia?	-Yes -No -I am skeptical
(19) If yes, how do you imagine the proposal for an ICT-based learning activity?	Open-ended question
(20) Do you think that virtual reality is suitable for this purpose?	-Yes -No -I am skeptical -I don’t know
(21) Do you think that augmented reality is suitable for this purpose?	-Yes -No -I am skeptical -I don’t know

**Table 2:** Round 2 statements and overall degree of agreement (expressing the percentage of “agree” and “strongly agree” replies on total number of responses excluding “I do not know” responses). Number of responses and percentages (in parentheses) are reported for each choice.

	Strongly Disagree *n* (%)	Disagree *n* (%)	Agree *n* (%)	Strongly Agree *n* (%)	I Do Not Know *n* (%)	Agreement (%)
(1) ICT technology can support the treatment of dyslexia as effectively as other methods can.	-	2 (13.33)	4 (26.67)	6 (40)	3 (20)	83.33
(2) ICT approaches can be seen as effective ways to integrate and, in some cases, substitute more traditional methods of treatment for developmental dyslexia.	-	-	7 (46.67)	6 (40)	2 (13.33)	89.27
(3) The main advantage of ICT approaches for the treatment of dyslexia is their flexibility, entailing the possibility to repeatedly propose the treatment several times per week, at the times that are more suitable for the children and their families.	-	2 (13.33)	2 (13.33)	9 (60)	2 (13.33)	87.69
(4) Other advantages of ICT trainings for dyslexia have to do with their ability to motivate and involve the children, and their ease of use. These characteristics allow children to work more with less effort.	-	1 (6.67)	1 (6.67)	6 (40)	7 (46.67)	90
(5) Among the advantages of ICT trainings there is cost-effectiveness, although it is not considered to be a prominent factor for the choice of the training to be proposed.	1 (6.67)	-	4 (26.67)	6 (40)	4 (26.67)	85.45
(6) ICT training should primarily address the processes involved in assembling the phonological structure of the words.	1 (7.69)	3 (23.08)	4 (30.77)	3 (23.08)	2 (15.38)	69.09
(7) Other, secondary goals for ICT training for dyslexia should focus on the improvement of both visual analysis and lexical retrieval abilities.	1 (6.67)	-	5 (33.33)	7 (46.67)	2 (13.33)	86.15
(8) Grapheme-to-phoneme (and vice-versa) conversion processes may be involved in the ICT training, but they should not be considered as prominent goals of the intervention.	2 (15.38)	5 (38.46)	2 (15.38)	1 (7.69)	3 (23.08)	50
(9) The optimal duration of the training should be between 2 and 6 months.	-	3 (20)	7 (46.67)	5 (33.33)	-	78.67
(10) The ideal time for the start of training with ICT tools is from the third year of primary school. In some cases, the start can be anticipated to the first or second year of primary school.	-	3 (20)	7 (46.67)	4 (26.67)	1 (6.67)	77.14
(11) The use of ICT training can contribute to sustaining motivation for learning in general.	-	2 (13.33)	3 (20)	7 (46.67)	3 (20)	85
(12) Augmented reality can be employed in the design of ICT trainings for dyslexia, but it should not play a prominent role.	1 (6.67)	2 (13.33)	5 (33.33)	2 (13.33)	5 (33.33)	70
(13) Trainings based on augmented reality could be introduced starting from 7–8 years of age.	1 (6.67)	-	6 (40)	5 (33.33)	3 (20)	83.33
(14) Augmented reality could be used to enhance the salient characteristics of the stimuli to be processed.	-	-	5 (33.33)	7 (46.67)	3 (20)	91.67
(15) Augmented reality could be used to provide a multi-sensory, multi-modal environment during the tasks, enriching the quality and quantity of information regarding the stimuli.	-	-	6 (40)	6 (40)	3 (20)	90
(16) Augmented reality could be used to highlight the difficult aspects of the stimuli to be processed, so that the child is alerted and ready to activate and focus her/his resources during the task.	1 (6.67)	1 (6.67)	2 (13.33)	6 (40)	5 (33.33)	82
(17) Augmented reality could be used to provide additional information for specific stimuli, according to the needs and requests of the child.	-	-	6 (40)	6 (40)	3 (20)	90
(18) Augmented reality could be used to add motivating elements to repetitive, boring tasks to make them more appealing.	-	1 (6.67)	5 (33.33)	8 (53.33)	1 (6.67)	88.57
(19) Augmented reality could facilitate automatization of metaphonological skills by highlighting processing units in the words (phonemes, syllables, and whole words).	-	2 (13.33)	5 (33.33)	4 (26.67)	4 (26.67)	80
(20) Further applications of augmented reality could be favoring attentional focusing and shifting processes.	-	-	7 (46.67)	4 (26.67)	3 (20)	83.33
(21) Additional applications of augmented reality in support of dyslexia extend to facilitating reading in everyday life contexts.	-	1 (6.67)	6 (40)	3 (20)	4 (26.67)	78.18
(22) Virtual reality can be employed in the design of ICT tools for the treatment of dyslexia.	-	2 (13.33)	7 (46.67)	4 (26.67)	1 (6.67)	77.14
(23) Training based on virtual reality could be introduced starting from 7–8 years of age.	-	1 (6.67)	6 (40)	5 (33.33)	3 (20)	85
(24) Virtual reality could be used to propose study subjects in realistic contexts, emphasizing the links between these subjects and real life.	-	-	5 (33.33)	6 (40)	4 (26.67)	90.91
(25) Virtual reality could be used to provide tasks embedded in ecologically plausible and varying contexts, thus fostering generalization processes.	-	-	6 (40)	6 (40)	3 (20)	90
(26) Virtual reality could be used to work on the child’s difficulties in a structured way through engaging, motivating tasks, and games.	-	-	8 (53.33)	4 (26.67)	3 (20)	86.67
(27) Virtual reality could be used to train learned skills through simulations and role-playing activities.	-	-	6 (40)	6 (40)	3 (20)	90
(28) Virtual reality could be used to design integrated trainings involving reading as well as visual and motor functions simultaneously.	-	-	5 (33.33)	6 (40)	4 (26.67)	90.91
(29) Virtual reality could facilitate automatization of metaphonological skills, lexical access, and perceptual discrimination.	-	1 (6.67)	5 (33.33)	3 (20)	5 (33.33)	78
(30) Further applications of virtual reality could aim at improving attentional processes and executive functions.	-	-	7 (46.67)	5 (33.33)	2 (13.33)	84.62
(31) Additional applications of virtual reality could extend to training more effective management of negative emotions related to dyslexia and learning difficulties.	-	2 (13.33)	3 (20)	4 (26.67)	5 (33.33)	76
(32) While using ICT tools for the treatment of dyslexia, maximum attention should be devoted to avoiding the risk of addiction.	-	4 (26.67)	3 (20)	7 (46.67)	1 (6.67)	78.57
(33) Use of ICT tools for dyslexia treatment should be proposed only after checking that adequate devices, connections, and familial support are available to the users.	-	-	2 (13.33)	13 (86.67)	-	97.33
(34) Use of ICT tools for the treatment of dyslexia should always be monitored by human supervisors who also ensure that the child’s needs, opinions, and feelings are taken into account.	-	-	-	15 (100)	-	100
(35) Use of ICT tools should be designed as to provide activities that are not only engaging, but also meaningful for the children/teenagers with dyslexia.	-	-	2 (13.33)	12 (80)	1 (6.67)	97.14
(36) ICT tools, including virtual and augmented reality, can also be used to support learning of school contents in children/teenagers with dyslexia.	-	-	5 (33.33)	6 (40)	4 (26.67)	90.91
(37) Support of general content learning in students with dyslexia could be achieved through ad-hoc activities with increasing levels of difficulty and complexity, emphasizing real understanding, and assimilation of meanings.	-	-	4 (26.67)	9 (60)	2 (13.33)	93.85
(38) ICT tools for students with dyslexia could provide training for web-surfing and searching skills, and for creative, responsible use of internet sources and tools.	-	1 (6.67)	3 (20)	6 (40)	5 (33.33)	88
(39) ICT tools could support general learning in students with dyslexia by providing a series of ordered activities where organization of study materials is required, based on the integration of both (possibly facilitated) reading and other, multi-media sources of information.	-	1 (6.67)	4 (26.67)	7 (46.67)	3 (20)	88.33

Red figures indicate that the criterion of 75% agreement was not reached.

**Table 3:** Agreement ratings for the three statements added in Round 3 and the different ratings collected at Round 2 and Round 3. Number of responses and percentages (in parentheses) are reported for each choice.

		Strongly Disagree *n* (%)	Disagree *n* (%)	Agree *n* (%)	Strongly Agree *n* (%)	I Do Not Know *n* (%)	Agreement (%)
**Statement 6**							
Round 2	ICT trainings should address primarily the processes involved in assembling the phonological structure of the words.	1 (7.69)	3 (23.08)	4 (30.77)	3 (23.08)	2 (15.38)	69.09
Round 3	ICT trainings may address the processes involved in assembling the phonological structure of the words.	0	1 (9.09)	4 (36.36)	3 (27.27)	3 (27.27)	82.5
**Statement 8**							
Round 2	Grapheme-to-phoneme (and vice-versa) conversion processes may be involved in the ICT training, but they should not be considered as prominent goals of the intervention.	2 (15.38)	5 (38.46)	2 (15.38)	1 (7.69)	3 (23.08)	50
Round 3	Grapheme-to-phoneme (and vice-versa) conversion processes may be involved in the ICT training.	-	1 (9.09)	4 (36.36)	5 (45.45)	1 (9.09)	86
**Statement 3b**							
Round 3	A further advantage linked to flexibility is the possibility to implement algorithms adapting the requests to the level of performance.	-	-	5 (45.45)	6 (54.55)	-	90.91

## Data Availability

The datasets have been deposited on Zenodo (doi:10.5281/zenodo.5572643) and are publicly accessible.
